# Increased serum fibroblast growth factor 21 levels are associated with adverse clinical outcomes after intracerebral hemorrhage

**DOI:** 10.3389/fnins.2023.1117057

**Published:** 2023-05-05

**Authors:** Keyang Chen, Wenting Huang, Jing Wang, Huiqin Xu, Lixin Ruan, Yongang Li, Zhen Wang, Xue Wang, Li Lin, Xiaokun Li

**Affiliations:** ^1^Department of Neurology, The Second Affiliated Hospital and Yuying Children’s Hospital of Wenzhou Medical University, Wenzhou, China; ^2^School of Pharmaceutical Sciences, Wenzhou Medical University, Wenzhou, China; ^3^Research Units of Clinical Translation of Cell Growth Factors and Diseases Research, Chinese Academy of Medical Science, Wenzhou Medical University, Wenzhou, China; ^4^Department of Neurology, The First Affiliated Hospital Hospital of Wenzhou Medical University, Wenzhou, China; ^5^The People’s Hospital of Pingyang, Wenzhou, China; ^6^The First People’s Hospital of Wenling, Taizhou, China

**Keywords:** fibroblast growth factor-21, prognosis, predictive value, intracerebral hemorrhage, enhancement

## Abstract

**Introduction:**

Intracerebral hemorrhage (ICH) is the most prevalent cause of death. We sought to explore whether serum Fibroblast growth factor 21 (FGF21) is of substantial benefit in predicting poor prognosis in ICH patient.

**Methods:**

A prospective, multicenter cohort analysis of serum FGF21 levels in 418 ICH patients was carried out. At three months following ICH start, the primary endpoint was death or major disability, whereas the secondary endpoint was death. We investigated the association between serum FGF21 and clinical outcomes. We added FGF21 to the existing rating scale to assess whether it enhanced the prediction ability of the original model. Effectiveness was determined by calculating the C-statistic, net reclassification index (NRI), absolute integrated discrimination improvement (IDI) index.

**Results:**

Among 418 enrolled patients, 217 (51.9%) of the all subjects had death or significant disability. Compared with patients in the lowest quartile group, those in the first quartile group had higher risk of the primary outcome (Odds ratio, 2.73 [95%CI,1.42–5.26, *p* < 0.05]) and second outcome (Hazard ratio, 4.28 [95%CI,1.61–11.42, *p* < 0.001]). The integration of FGF21 into many current ICH scales improved the discrimination and calibration quality for the integrated discrimination index’s prediction of main and secondary findings (all *p* < 0.05).

**Conclusion:**

Elevated serum FGF21 is associated with increased risks of adverse clinical outcomes at 3 months in ICH patients, suggesting FGF21 may be a valuable prognostic factor.

## Introduction

More than 10% of all stroke hospitalizations are due to spontaneous intracerebral hemorrhage (ICH), the second most common kind of stroke (Sacco et al., 2009). It’s considered a major issue since it causes 40% death and significant disability rates, making it a significant public health concern (Qureshi et al., 2001, 2009). Hence, rehabilitation management for ICH patients must be guided by accurate prognoses and risk stratification. The original ICH score was the first commonly used and straightforward clinical grading system for ICH (Hemphill et al., 2001). New predictive ICH models and enhancements to the original ICH score (Gregório et al., 2018) have been implemented, such as the FUNC score (Rost et al., 2008), ICH-FOS score (Ji et al., 2013). However, none of these has achieved widespread acceptance and utilization in actual clinical practice (Schmidt et al., 2018). In recent decades, predictors of ICH prognostic models have been enhanced by adding novel laboratory indicators to clinical risk variables in order to increase the prediction capacity of the model (Cai et al., 2021).

Fibroblast growth factor 21 (FGF21) is a protein that is primarily produced in the liver and has many effects on how tissues balance their energy. It belongs to the subfamily of fibroblast growth factors (Chen et al., 2022). As it lacks heparin-binding domains, it is secreted into the bloodstream and exerts its endocrine effects there. It has been suggested that FGF21, a key regulator of glucose and lipid metabolism, might be used to treat metabolic illnesses (Geng et al., 2020). Furthermore, several epidemiological studies have found that FGF21 helps predict prognosis in cardiovascular disease and atherosclerosis (Chou et al., 2016; Li et al., 2016; Tabari et al., 2019). Nonetheless, significant population-based studies on the prognostic usefulness of serum FGF21 on functional outcome and death in patients with ICH are lacking. In light of this, the current study sought to evaluate the relationship between blood FGF21 levels and prognosis in ICH patients.

## Materials and methods

### Ethical statements

We performed this investigation utilizing data from a multicenter, prospective registry study done in five hospitals in the southeast coastline region of Zhejiang Province, China. For this predetermined biomarker analysis, blood samples were taken from study locations. The study was authorized by the institutional review boards of all research facilities, and we acquired written informed permission to participate in this study from legal representatives of patients. The study is registered at http://www.chictr.org.cn (ChiCTR2100051104).

### Study population

The Stroke and Fibroblast Growth Factor Biomarkers Cohort Study (SFBCS) was established to investigate the determinants of stroke and prognosis in the coastal area of southeast Zhejiang Province, China. The SFBCS was a multicenter, prospective observational study of stroke biomarkers and outcomes that recruited 1,510 stroke patients (including ischemic stroke, transient ischemic attack, and primary ICH) within 24 h of symptom onset, from March 1, 2021, to August 31, 2022. With the exception of ischemic stroke and transient ischemic attack, this study included all patients over the age of 18 in our cohort who had ICH (International Classification of Diseases, Tenth Revision, I61) during their hospital stay.

Each facility reviews suspected ICH patients’ electronic medical records upon admission from the emergency room and the neurology/neurosurgery departments to rule out survival bias. Almost every patient had a clinically meaningful blood sample that could be tested in the laboratory. Hemorrhagic infarcts, traumatic hemorrhages, and hemorrhagic conversion of a recent ischemic stroke were excluded, as were patients with ICH caused by malignancies, irreversible coagulopathy, Dural venous sinus thrombosis, vascular malformations, aneurysms, tumors, or other causes.

### Data collection

Age, sex, history of hypertension, diabetes, hyperlipidemia, atrial fibrillation, smoking, alcohol intake, were included in demographic and clinical information. One cigarette per day was classified as smoking. Drinking at least 80 grams of liquor per day was defined as alcohol usage. Hypertension and diabetes were identified based on self-reported medical histories. At admission, the Glasgow Coma Scale (GCS), the National Institutes of Health Stroke Scale (NIHSS), and the initial post-stroke systolic (SBP) and diastolic (DBP) blood pressure (mmHg) were measured. Laboratory data collection (blood glucose, blood lipids, blood creatinine, etc.) was conducted in each participating hospital. In ICH patients, baseline radiological data from brain CT or MRI scans were collected. The location of intracerebral hemorrhage was categorized into lobar (frontal, temporal, parietal, and occipital); deep (lenticular or caudate nucleus, thalamus, internal or external capsule); brainstem; and cerebellum. The volume of the hemorrhage was calculated according to the ABC/2 method (Kothari et al., 1996). Based on the CT scan, hematoma expansion was calculated as a threshold >33% or >6 mL (Dowlatshahi et al., 2011) and was analyzed as a binary variable. All images were assessed by one board-certified radiologist and were blinded to clinical data.

Blood samples were collected from the vein immediately upon admission within 24 h, the 3rd and 7th day after the diagnosis. We calculated the cumulatively averaged FGF21 concentration using available FGF21 measurements to capture short-term longitudinal FGF21 patterns of participants. All samples were frozen at −80°C refrigerator in the central laboratory of Wenzhou Medical University until laboratory testing. The serum levels of FGF21 were determined using enzyme-linked immunosorbent assays (ELISAs) according to the manufacturer’s instructions. Routine blood analyses were performed in the hospital’s central laboratory.

### Functional outcome assessment

Patient’s follow-up at 90-day was conducted by qualified personnel or physicians through telephone or in-person interview following a defined technique. Functional effects after ICH were evaluated using the modified Rankin Scale (mRS) with a range between 0 and 6, where a number of 0 means no illness and a number of 6 means death. At follow-up, the primary endpoints were composite events (mRS score 3–6), which comprised death from any cause (mRS score 6) or severe disability (mRS score 3–5). The secondary outcome was all-cause death.

### Statistical analysis

All ICH patients were classified according to quartiles of FGF21 levels. Using the χ^2^ test for categorical data (such as sex and medical history) and ANOVA or the Kruskal-Wallis test for continuous variables (such as age), the baseline characteristics of patients were compared across quartiles of FGF21. Categorical variables are expressed as percentages, whereas continuous variables are represented as the mean (SD) or median (IQR). Missing data were imputed using the multiple imputation of chained equations approach. Utilizing univariable and multivariable analysis with logistic or Cox regression based on clinical confounding risk variables from clinical experience and literature, the association between FGF21 and adverse outcomes of ICH was evaluated. Comparing the high FGF21 quartiles to the low FGF21 quartiles, odds ratios (ORs) and 95% confidence intervals (CIs) for the primary or secondary outcome were calculated. Finally, Model 1 was adjusted for age (years) and sex (male or female); Model 2 was adjusted for age, sex, history of hypertension (yes or no), diabetes (yes or no), stroke (yes or no), and prior mRS (categorical); Model 3 was additionally adjusted for SBP (continuous), GCS score (continuous), location of hematoma (categorical), hematoma volume (continuous), and hematoma expansion (categorical). Kaplan–Meier survival curve was performed to show the cumulative incidence of death, where a log-rank test was stratified by FGF21 quartile. In addition, we assessed the predictive performance of the ICH score, ESSCN-ICH score, FUNC score, and ICH-FOS score, respectively. We appended FGF 21 to the four scales and calculated the difference in model performance using C statistics, the category-free net reclassification index (NRI), and integrated discrimination improvement (IDI). NRI and IDI are two alternatives to AUC for evaluating the performance improvement and usability of a new model (Pencina et al., 2008). To test the robustness of our findings, we conducted subgroup analyses stratified by sex, age, history of hypertension and diabetes, current smoking, and alcohol consumption. All data were analyzed with SAS version 9.4 software (SAS Institute Inc., Cary, NC) and R statistical software (Harrell Miscellaneous) version 4.2.1. *p* < 0.05 (two-sided) was considered statistically significant.

## Results

### Baseline characteristics

**Figure 1:**
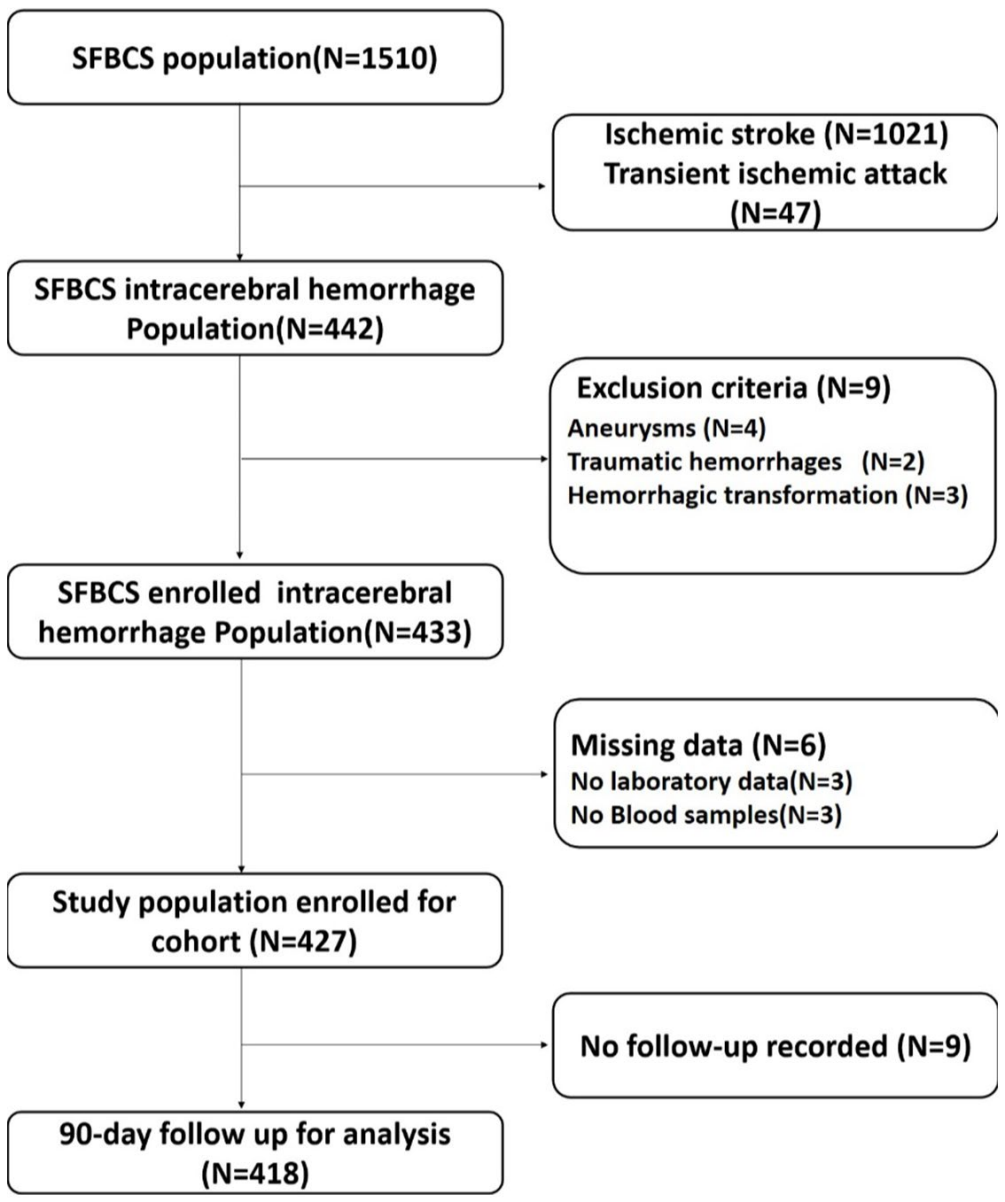
Flow chart of patient selection. SFBCS indicates stroke and fibroblast growth factor biomarkers cohort study.

The final pooled cohort consisted of 418 patients (Figure 1). At 90-day, 86 (20.6%) of patients had died and 217 (51.9%) had an unsatisfactory outcome (mRS 3 to 6). The mean (SD) age was 62.3 (12.7) years, and 271 (64.8%) participants were male. Table 1 displays baseline characteristics by quartiles of FGF21. Patients with higher serum FGF21 were more likely to be older, have higher blood glucose, and have lower high-density lipoprotein than those with lower serum FGF21. Based on the neurological examination and radiological parameters upon admission, patients in the upper quartiles of FGF21 had a greater NIHSS score and a larger hematoma volume but lower GCS score.

**Table 1:** Baseline characteristics of all enrolled patients according to FGF21 quartiles.

		FGF21, pg./mL				
Characteristic	All population	<141.9	141.9–274.2	274.2–489.5	≥489.5	*p* for trend
No.	418	105	104	105	104	
Age, years, mean ± SD	62.3 (12.7)	61.4 (12.0)	60.4 (12.3)	62.4 (12.0)	65.1 (14.1)	0.019
Male, *n* (%)	271 (64.8)	64 (61.0)	68 (65.4)	71 (67.6)	68 (65.4)	0.456
BMI	23.9 (3.48)	23.8 (4.21)	24.1 (3.30)	23.9 (3.33)	23.8 (2.99)	0.899
Medical history, *n* (%)						
Hypertension	328 (78.5)	84 (80.0)	77 (74.0)	84 (80.0)	83 (79.8)	0.767
Diabetes	57 (13.6)	13 (12.4)	15 (14.4)	12 (11.4)	17 (16.3)	0.555
Prior stroke	50 (12.0)	10 (9.52)	15 (14.4)	17 (16.2)	8 (7.69)	0.800
Coronary artery disease	10 (2.39)	3 (2.86)	0 (0.00)	2 (1.90)	5 (4.81)	0.250
Atrial fibrillation	12 (2.87)	2 (1.90)	1 (0.96)	5 (4.76)	4 (4.85)	0.188
Smoking	115 (27.5)	25 (23.8)	28 (26.9)	31 (29.5)	31 (29.8)	0.292
Alcohol drinking	129 (30.9)	29 (27.6)	35 (33.7)	32 (30.5)	33 (31.7)	0.649
Laboratory data						
FPG, mmol/L	6.50 (5.47–8.10)	5.83 (5.23–7.29)	6.20 (5.26–7.55)	6.63 (5.60–8.27)	7.13 (5.89–8.81)	<0.001
TG, mmol/l	1.21 (0.83–1.77)	1.09 (0.76–1.56)	1.25 (0.85–1.83)	1.22 (0.86–1.76)	1.23 (0.81–1.79)	0.313
TC, mmol/l	4.32 (1.04)	4.35 (0.87)	4.20 (1.02)	4.49 (1.11)	4.25 (1.13)	0.928
LDL-C, mmol/l	2.68 (2.03–3.35)	2.72 (2.11–3.38)	2.60 (1.88–3.21)	2.71 (2.26–3.48)	2.68 (1.98–3.21)	0.974
HDL-C, mmol/l	1.08 (0.91–1.33)	1.18 (0.98–1.38)	1.06 (0.91–1.31)	1.11 (0.88–1.34)	1.02 (0.84–1.28)	0.018
eGFRcr, mL/min/1.73 m^2^	134 (108–159)	135 (111–158)	134 (113–158)	137 (107–160)	128 (98.8–160)	0.293
Clinical severity						
Initial NIHSS score, (IQR)	8 (3–16)	4 (1–10)	7 (4–13)	9 (3–23)	13 (6–29)	<0.001
Glasgow Coma Scale score, (IQR)	15 (11–15)	15 (15–15)	15 (14–15)	15 (10–15)	13 (7–15)	<0.001
ICH location, *n* (%)						0.409
Lobar	79 (18.9)	22 (21.0)	12 (11.5)	14 (13.3)	31 (29.8)	
deep	286 (68.4)	68 (64.8)	78 (75.0)	81 (77.1)	59 (56.7)	
Brainstem	26 (6.22)	10 (9.52)	6 (5.77)	5 (4.76)	5 (4.81)	
cerebellum	27 (6.46)	5 (4.76)	8 (7.69)	5 (4.76)	9 (8.65)	

Continuous variables are expressed as mean ± SD or as median (inter quartile range). Categorical variables are expressed as frequency (%). mRS, modified Rankin Scale; NIHSS, National Institutes of Health Stroke Scale; BMI, body mass index; FPG, fasting plasma glucose; TG, triglycerides; TC, total cholesterol; LDL-C, low density lipoprotein cholesterol; HDL-C, high density lipoprotein cholesterol; ICH, intracranial hemorrhage.

### Association between FGF21 and ICH prognosis

**Figure 2:**
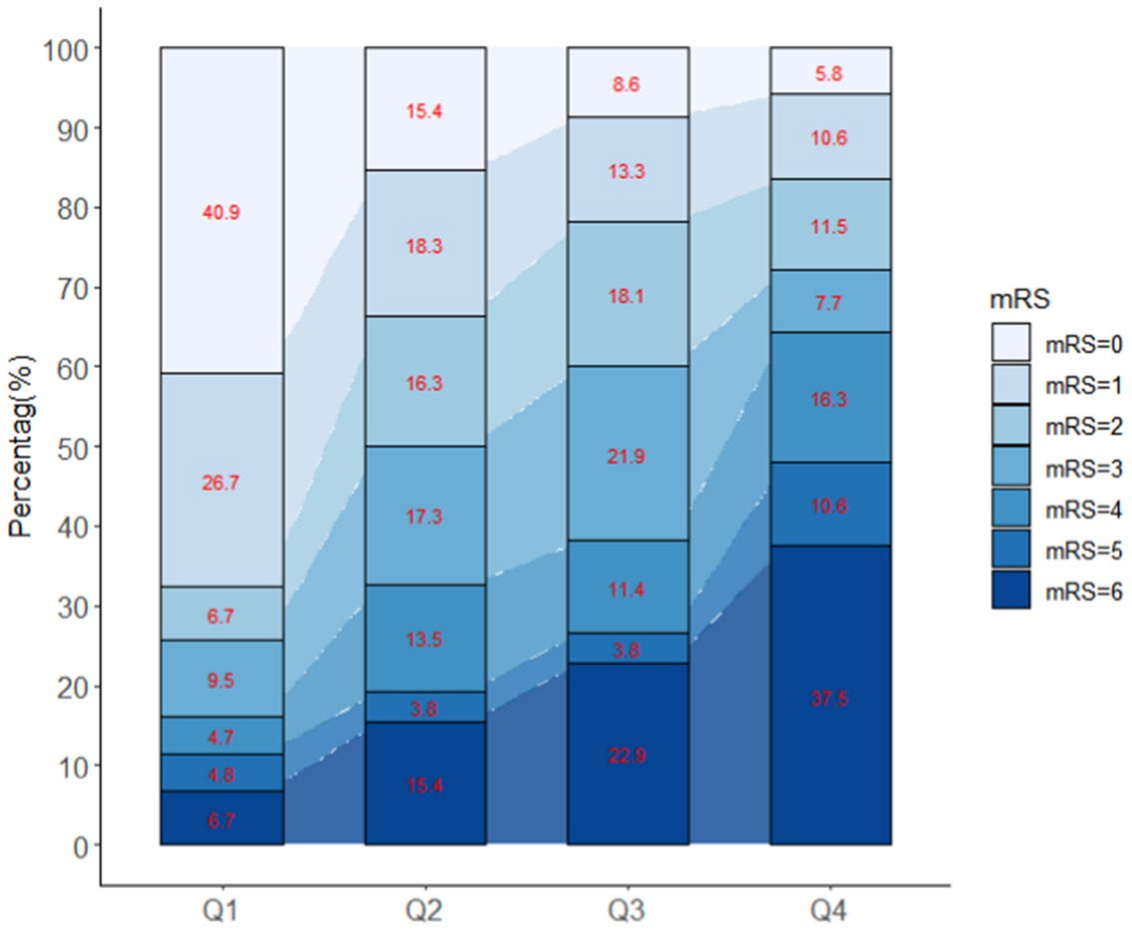
Distribution of mRS according to different quartiles of FGF21.

**Figure 3:**
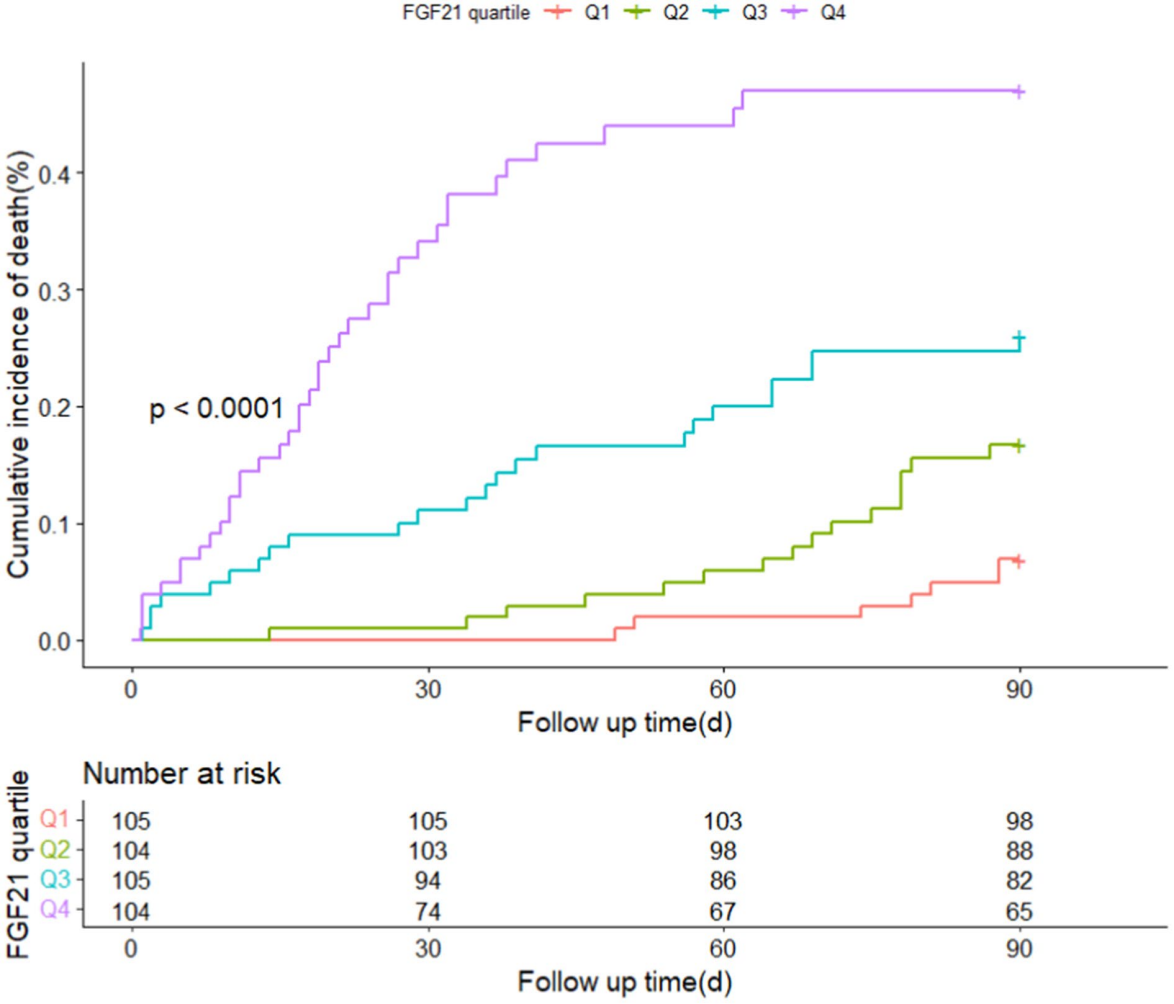
Cumulative incidence of 90-day death stratified by quartiles of FGF21.

Patients with different quartiles of FGF21 varied in the distribution of mRS (Figure 2). The cumulative incidence rates of primary outcome at 3 months from the lowest quartile to the highest quartile of serum FGF21 were 25.7, 50.0, 60.0, and 72.1%, respectively. After adjustment for age, sex, history of hypertension, diabetes, stroke, prior mRS, SBP, GCS, hematoma location, hematoma volume, and hematoma expansion, the multivariable logistic regression analyses remained a significant association between FGF21 and unfavorable clinical outcomes [OR, 3.38 (95% CI, 1.41–8.06, *p* < 0.001); Table 2]. In subgroup analyses stratified by sex, age, current smoking, alcohol consumption, history of hypertension and diabetes, no significant interaction between serum FGF21 levels and these prespecified factors was observed (all *p* for interaction >0.05) and a substantial connection of FGF21 with risk of the primary outcome was seen in every subgroup (Supplementary Figure S1). Multivariable analyses between FGF21 and death seemed to have a relationship, however, after controlling all confounding variables, the connection was not significant (HR, 3.42 [95%CI, 0.65–17.93, *p* > 0.05]) (Supplementary Tables S1 in the Data Supplement). In addition, the cumulative incidence of 90-day death increased in higher FGF21 quartile groups (*p* < 0.001, log-rank test; Figure 3).

**Table 2:** Multivariable analyses of FGF21 to predict poor outcomes according to quartiles at baseline.

	Events, *N* (%)	Unadjusted	Model 1	Model 2	Model 3
		OR (95% CI)	OR (95% CI)	OR (95% CI)	OR (95% CI)
Primary outcome: death or major disability (mRS 3–6)					
Q1	27 (25.7)	Reference	Reference	Reference	Reference
Q2	52 (50.0)	2.89 (1.63–5.23)*	2.96 (1.66–5.37)*	2.20 (1.06–4.73)*	2.27 (1.02–5.06)*
Q3	63 (60.0)	4.34 (2.43–7.89)*	4.53 (2.53–8.29)*	4.16 (1.92–9.36)*	3.15 (1.40–7.08)*
Q4	75 (72.1)	7.47 (4.10–14.01)*	7.84 (4.27–14.83)*	4.62 (2.12–10.51)*	3.38 (1.41–8.06)*

Model 1: adjusted for age, sex. Model 2: Model 1 + history of hypertension, diabetes, dyslipidemia, prior mRS. Model 3: Model 2 + systolic blood pressure, Glasgow Coma Scale score, hematoma location, hematoma volume. Abbreviations: CI, confidence interval; mRS, modified Rankin Scale; OR, odds ratio. *p < 0.05.Q: quartile.

### The inclusion of FGF21 enhances a model for predicting adverse results

We discovered whether adding serum FGF21 to the different recognized scales improved the risk prediction of adverse clinical outcomes with ICH (Table 3). Adding serum FGF21 to four prognostic scales (ICH, ESSCN-ICH, FUNC, ICH-FOS) considerably ameliorated their C-statistic performance (all *p* < 0.05). The range of NRI was from 5.91 to 13.35% in our study, which indicated that the addition of the serum FGF21 promotes the four recognized scores’ predictive performance for the primary outcome. For instance, the NRI of ESSCN-ICH plus FGF21 was 5.91% (0.39–11.44); the NRI of ICH-FOS score plus FGF21 was 9.40% (3.15–15.64); the NRI of FUNC score plus FGF21 was 13.35 (4.90–21.78). The NRI of ICH score plus FGF21 was 10.96% (4.14–17.79). The predictive ability of the new model assessed by IDI in our study can be clarified similarly to the explanation of NRI. For predictive ability of death, the performance of ICH score was significantly improved by adding serum FGF21 (C-statistics: 0.936 vs. 0.916, *p* = 0.003); (NRI = 6.89%, *p* = 0.033); (IDI =2.91%, *p* = 0.016). Adding serum FGF21 to FUNC score did significantly improve discriminatory power but did not improve risk reclassification for the death (NRI = −0.26%, *p* = 0.947; IDI =3.88%, *p* = 0.003) (Supplementary Table S2). However, no significant improvements were observed after the addition of serum FGF21 to the ESSCN-ICH, ICH-FOS scales (NRI and IDI, all *p* > 0.05) (Supplementary Table S2).

**Table 3:** Reclassification and discrimination statistics for 90-day outcomes by FGF21 among patients with ICH.

	C-statistic		Category-free NRI		IDI	
	Estimate (95% CI)	*p* value	Estimate (95% CI), %	*p* value	Estimate (95% CI), %	*p* value
Primary outcome: death or major disability (mRS 3–6)						
ICH score	0.761	Reference		Reference		Reference
ICH + FGF21	0.810 (0.770–0.850)	<0.001	10.96 (4.14–17.79)	0.002	4.63 (2.61–6.65)	<0.001
FUNC score	0.737	Reference		Reference		Reference
FUNC+FGF21	0.782 (0.739–0.825)	0.001	13.35 (4.90.-21.78)	0.002	5.35 (3.16–7.53)	<0.001
ESSEN-ICH score	0.847	Reference		Reference		Reference
ESSEN-ICH + FGF21	0.864 (0.831–0.897)	0.004	5.91 (0.39–11.44)	0.035	1.82 (0.58–3.07)	0.004
ICH-FOS	0.840	Reference		Reference		Reference
ICH-FOS + FGF21	0.855 (0.819–0.889)	0.029	9.40 (3.15–15.64)	<0.001	2.09 (0.73–3.46)	0.003

ICH score, Intracerebral Hemorrhage score; FUNC score, Functional Outcome score; ESSEN-ICH score, Essen Stroke Risk Intracerebral Hemorrhage score; mRS, Modified Rankin Scale; IDI, Integrated Discrimination Improvement, NRI, Net Reclassification Improvement.

## Discussion

This is the first research to shed light on the association between the level of FGF21 and ICH prognosis. In this prospective multicenter study, we established a significant association between elevated serum FGF21 levels and the composite outcome of death or major disability in ICH patients, and the association remained an independent risk factor even after adjusting for potential ICH risk factors.

FGF21 controls energy metabolism and functioned as a stress hormone in humans (Salminen et al., 2017). Several population-cohort studies focused on the role of FGF21 in diverse diseases’ prognosis. In a national multicenter cohort study, circulating FGF21 levels are inversely connected with muscular strength but are not independently correlated with muscle mass (Roh et al., 2021). Independently, the FGF21/adiponectin ratio predicted the emergence of pre-diabetes and diabetes in a prospective Shanghai Nicheng cohort study (Liu et al., 2021). Higher blood FGF21 levels were related to worse survival in hepatocellular carcinoma patients, according to a cohort study from Guangdong province (Liu et al., 2022). Additionally, Ong et al. (2019) and Wu et al. (2022) revealed that the amount of serum FGF21 was linked with the prognosis of several cardiovascular illnesses. Moreover, only one cohort study evaluated the prognostic value of serum FGF21 among patients with acute ischemic stroke (Zheng et al., 2021). Nevertheless, serum FGF21 and its association with unfavorable outcomes have yet to be assessed in a prospective multicenter cohort with ICH. In our population of Chinese patients with ICH, we discovered that higher levels of FGF21 were strongly related to worse long-term endpoints, even after controlling for established risk variables.

We added FGF21 to the prognostic models for predicting clinical outcomes in patients with ICH. Verified by C-statistic, IDI, and NRI across various prognostic ICH scales, the predictive performance of FGF21 for adverse outcomes at 90-day follow-up was enhanced. The development of scoring systems for ICH patients has gained momentum in the past and is still ongoing, such as the ICH score, Essen-ICH score, FUNC score, and ICH-FOS score from China. Until now, the ICH score (Wang et al., 2013), Essen-ICH score, and FUNC score have been validated in the Chinese population (Suo et al., 2018). However, they are limited by ignoring metabolic and physiologic blood parameters on patient’s initial arrival. The ICH-FOS demonstrated superior discrimination over existing ICH models for 3-month mortality and negative functional outcomes (Ji et al., 2022). Similar performance for predicting unfavorable outcomes and death was observed in our study. Meanwhile, with the addition of FGF21, the prognosis value of four scales was promoted for unfavorable functional outcome or death. Further studies are warranted to validate these findings and to explore the biological possible mechanism of FGF21 in ICH.

The direct targets and mechanisms linking FGF21 to unfavorable outcomes in ICH patients are still elusive. Traumatic mechanical compression from a hematoma, thrombin production, inflammation, excitotoxicity, and oxidative stress are only some of the secondary injuries that contribute to the overall pathological damage seen during ICH (Keep et al., 2012). Due to its extremely low heparin-binding affinity, FGF21 can pass the blood–brain barrier and control energy balance, glucose, and lipid homeostasis (Hsuchou et al., 2007). FGF21 protects tissues from increased oxidative stress damage, also increases its circulating concentration (Gómez-Sámano et al., 2017). Leng et al. (2015) focused on the neuroprotective role of FGF21, indicated that FGF21 can protect primary neurons from glutamate-induced excitotoxicity. Moreover, FGF21 reduces neuroinflammation and oxidative stress through regulating two pathways (Kang et al., 2020). In addition, FGF21 is related to hypertension, an important risk factor for ICH (Semba et al., 2013). FGF21 plays an important role in preventing angiotensin II-induced hypertension and vascular dysfunction (Pan et al., 2018). Above all, a higher circulating FGF21 level may occur after ICH because of a greater demand for its protective effects throughout the damage process. Further investigations are needed to gain a better insight into the physiological mechanism between FGF21 and ICH.

There are several shortcomings to this study that warrant discussion. First, our study only includes admission and in-hospitalization characteristics, which typically predict short-term outcomes. Second, this study only included Chinese people and assessed 90-day outcomes, longer follow-up periods, such as one year or more, require additional research. Third, no big enough collective of participants enrolled in this study, selection bias should be pay attention to. Yet, we adopted a pursuit approach to enroll almost all ICH patients with mild to severe symptoms. Considering the observational nature of this study, the potential occurrence of residual confounding represents a fourth constraint.

## Conclusion

In conclusion, the present study provided evidence that elevated serum FGF21 was associated with unfavorable prognosis in ICH patients. Serum FGF21 may have potential prognostic value in risk stratification of ICH patients.

## Data availability statement

The original contributions presented in the study are included in the article/Supplementary material, further inquiries can be directed to the corresponding authors.

## Ethics statement

The studies involving human participants were reviewed and approved by institutional review boards of the Second Affiliated Hospital of Wenzhou Medical University.

## Author contributions

KC was involved in the design of the study, data collection, and manuscript writing. XL took part in the design of the study, was a recipient of the obtained funding. HX, LR, YL, JW, and ZW were involved in data collection. XW and WH took part in the statistical analysis. LL and XL were a recipient of the obtained funding and was involved in the interpretation of the data and the manuscript revision.

## Funding

This work was supported by Medical Health Science and Technology Project of Zhejiang Provincial Health Commission (No. 2023KY905; WKJ-ZJ-2130;2023KY307). The National Natural Science Foundation of China (No. 81971180).

## Conflict of interest

The authors declare that the research was conducted in the absence of any commercial or financial relationships that could be construed as a potential conflict of interest.

## Publisher’s note

All claims expressed in this article are solely those of the authors and do not necessarily represent those of their affiliated organizations, or those of the publisher, the editors and the reviewers. Any product that may be evaluated in this article, or claim that may be made by its manufacturer, is not guaranteed or endorsed by the publisher.
