# Supplementary material for: Increased serum fibroblast growth factor 21 levels are associated with adverse clinical outcomes after intracerebral hemorrhage
Source: Front Neurosci. 2023 May 5;17:1117057. doi: 10.3389/fnins.2023.1117057 (PMC10198380; doi:10.3389/fnins.2023.1117057)

**Supplement Table 1 Multivariable Analyses of FGF21 to Predict death according to quartiles at baseline**

|  | Unadjusted | Model 1 | Model 2 | Model 3 |
| --- | --- | --- | --- | --- |
| Death (mRS 6) | HR (95% CI) | HR (95% CI) | HR (95% CI) | HR (95% CI) |
| Q1 | Reference | Reference | Reference | Reference |
| Q2 | 2.55(1.04-6.89)* | 2.68(1.08-7.29)* | 2.09 (0.71-7.08) | 3.20 (0.56-18.18) |
| Q3 | 4.15(1.78-10.87)* | 4.50(1.91-11.91)* | 3.65 (1.25-12.34)* | 3.06 (0.52-18.04) |
| Q4 | 8.40(3.74-21.55)* | 9.17(4.02-23.84)* | 4.16 (1.45-13.95)* | 3.42 (0.65-17.93) |
| Model 1: adjusted for age, sex. Model 2: Model 1+history of hypertension, diabetes, dyslipidemia, prior mRS. Model 3:Model 2+systolic blood pressure, Glasgow Coma Scale score, hematoma location, hematoma volume. Abbreviations: CI, confidence interval- mRS, modified Rankin Scale- HR, Hazard ratio.* *P*<0.05.Q: quartile | | | | |

**Supplement Table 2 Reclassification and discrimination statistics for 90-day death by FGF-21**

|  | C-statistic |  | Category-free NRI |  | IDI |  |
| --- | --- | --- | --- | --- | --- | --- |
|  | Estimate (95% CI) | P value | Estimate (95%CI), % | P value | Estimate (95%CI), % | P value |
| Death |  |  |  |  |  |  |
| ICH score | 0.916 | Reference |  | Reference |  | Reference |
| ICH+FGF21 | 0.936(0.904-0.968) | 0.003 | 6.89 (0.54-13.25) | 0.033 | 2.91(0.55-5.27) | 0.016 |
| FUNC score | 0.921 | Reference |  | Reference |  | Reference |
| FUNC+FGF21 | 0.938(0.905-0.972) | 0.006 | -0.26 (-7.88-7.36) | 0.947 | 3.88 (1.36-6.39) | 0.003 |
| ESSEN-ICH score | 0.947 | Reference |  | Reference |  | Reference |
| ESSEN-ICH+FGF21 | 0.955(0.934-0.975) | 0.051 | -0.22(-6.05-5.61) | 0.942 | 1.41 (-0.41-3.23) | 0.130 |
| ICH-FOS score | 0.956 | Reference |  | Reference |  | Reference |
| ICH-FOS+FGF21 | 0.961(0.944-0.979) | 0.167 | 1.42(-2.12-4.97) | 0.432 | 2.08 (-3e-02-4.19) | 0.053 |
| ICH, intracerebral hemorrhage; FUNC, functional outcome score; ESSEN-ICH,Essen Stroke Risk Intracerebral hemorrhage Score;IDI, integrated discrimination improvement; mRS, modified Rankin Scale; NRI, net reclassification improvement. | | | | | | |

Figure S1

Subgroup analyses the association of FGF-21 with risk of the primary outcome


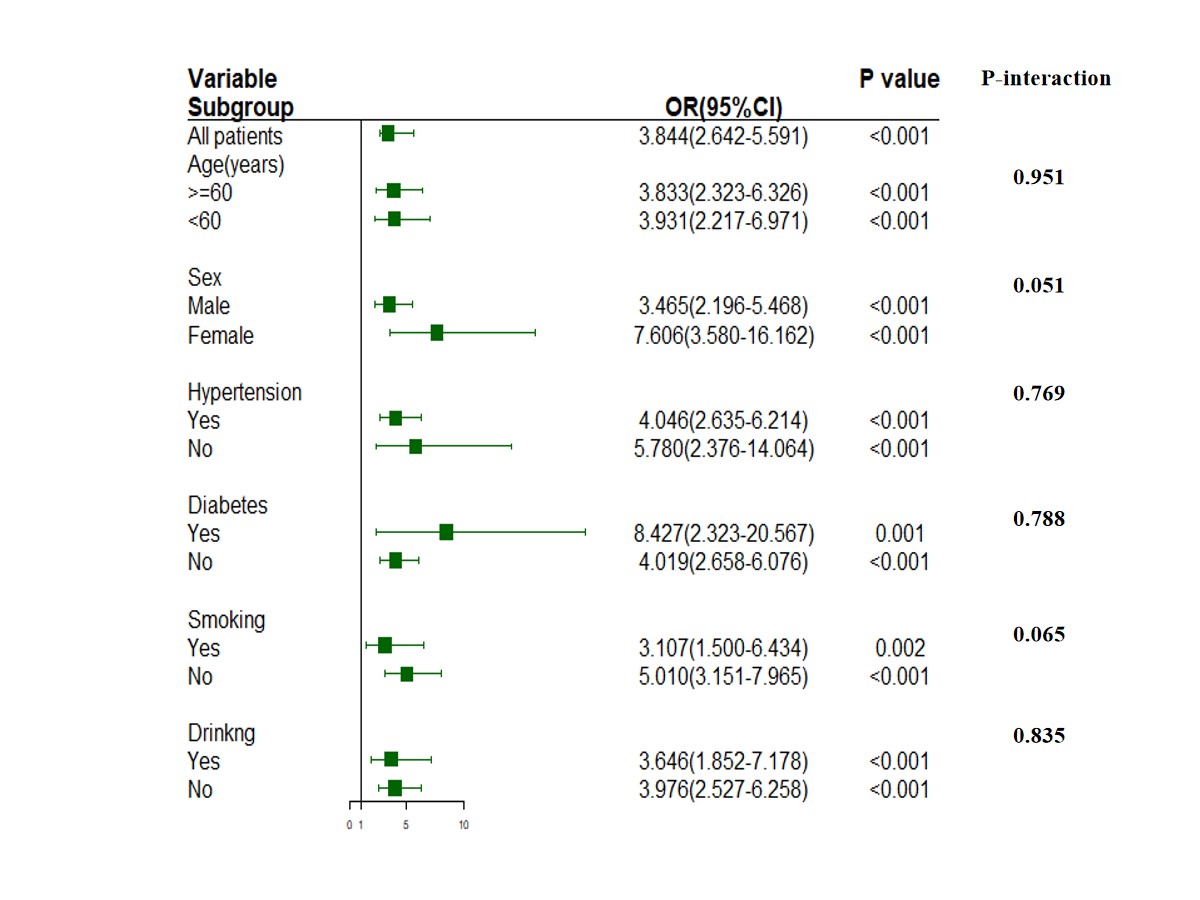


Figure S2

Subgroup analyses the association of FGF-21 with risk of the second outcome


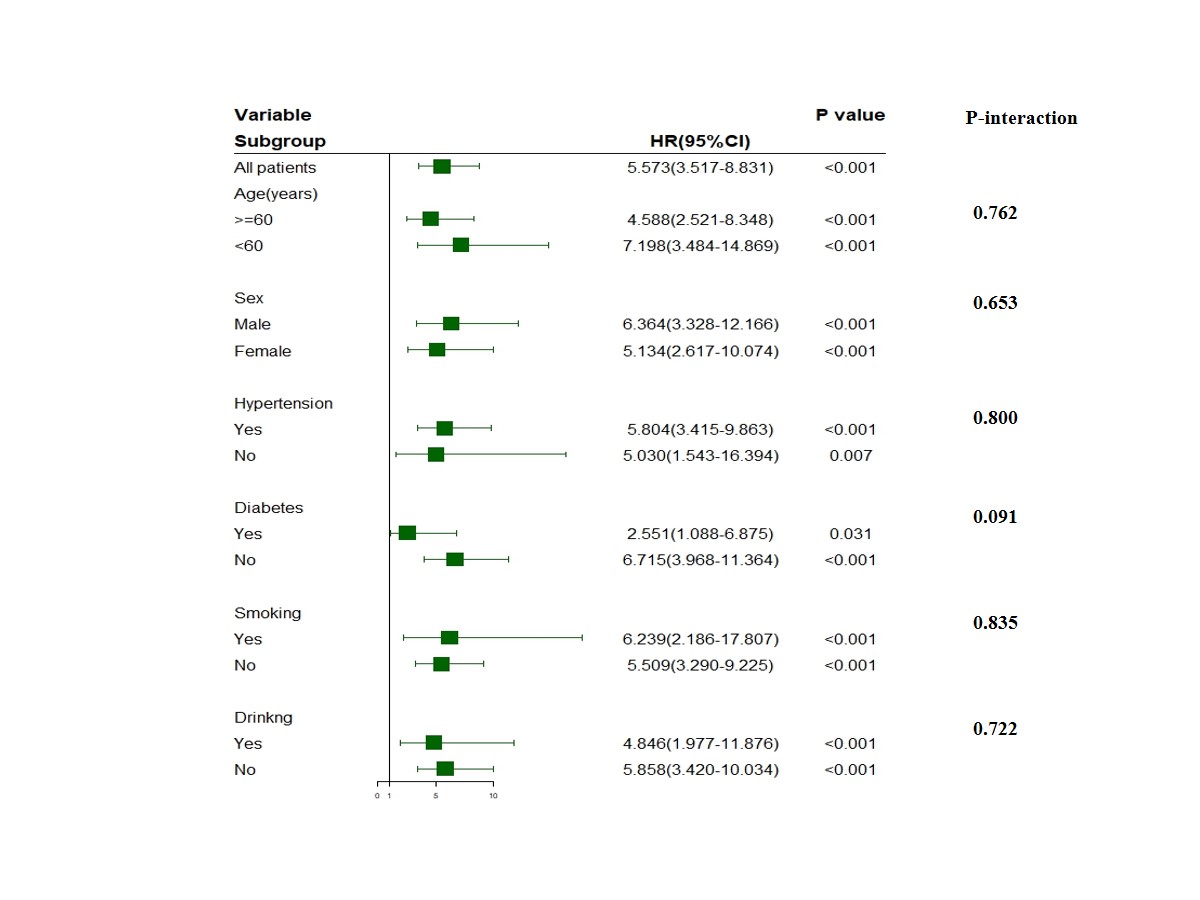

Supplement: Supplementary file 1 [file Data_Sheet_1.docx]
